# A niche for null models in adaptive resource management

**DOI:** 10.1002/ece3.8541

**Published:** 2022-01-13

**Authors:** David N. Koons, Thomas V. Riecke, G. Scott Boomer, Benjamin S. Sedinger, James S. Sedinger, Perry J. Williams, Todd W. Arnold

**Affiliations:** ^1^ Department of Fish, Wildlife, and Conservation Biology Graduate Degree Program in Ecology Colorado State University Fort Collins Colorado USA; ^2^ Department of Natural Resources and Environmental Science University of Nevada Reno Nevada USA; ^3^ Program in Ecology, Evolution, and Conservation Biology University of Nevada Reno Nevada USA; ^4^ Division of Migratory Bird Management U.S. Fish and Wildlife Service Laurel Maryland USA; ^5^ College of Natural Resources University of Wisconsin – Stevens Point Stevens Point Wisconsin USA; ^6^ Department of Fisheries, Wildlife and Conservation Biology University of Minnesota St. Paul Minnesota USA

**Keywords:** adaptive harvest management, climate change, forecast, knowledge, learning, persistence, prediction

## Abstract

As global systems rapidly change, our collective ability to predict future ecological dynamics will become increasingly important for successful natural resource management. By merging stakeholder objectives with system uncertainty, and by adapting actions to changing systems and knowledge, adaptive resource management (ARM) provides a rigorous platform for making sound decisions in a changing world. Critically, however, applications of ARM could be improved by employing benchmarks (i.e., points of reference) for determining when learning is occurring through the cycle of monitoring, modeling, and decision‐making steps in ARM. Many applications of ARM use multiple model‐based hypotheses to identify and reduce systematic uncertainty over time, but generally lack benchmarks for gauging discovery of scientific evidence and learning. This creates the danger of thinking that directional changes in model weights or rankings are indicative of evidence for hypotheses, when possibly all competing models are inadequate. There is thus a somewhat obvious, but yet to be filled niche for including benchmarks for learning in ARM. We contend that carefully designed “ecological null models,” which are structured to produce an expected ecological pattern in the absence of a hypothesized mechanism, can serve as suitable benchmarks. Using a classic case study of mallard harvest management that is often used to demonstrate the successes of ARM for learning about ecological mechanisms, we show that simple ecological null models, such as population persistence (*N_t_*_+1_ = *N_t_*), provide more robust near‐term forecasts of population abundance than the currently used mechanistic models. More broadly, ecological null models can be used as benchmarks for learning in ARM that trigger the need for discarding model parameterizations and developing new ones when prevailing models underperform the ecological null model. Identifying mechanistic models that surpass these benchmarks will improve learning through ARM and help decision‐makers keep pace with a rapidly changing world.

## Graphical abstract

Ecological null models based on sound logic can be used as benchmarks for learning in ARM that trigger the need for new model parameterizations when prevailing models underperform the ecological null model. Identifying mechanistic models that surpass these benchmarks will improve learning through ARM and help guide decision making in a rapidly changing world.

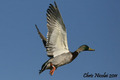

## 1 INTRODUCTION

Most ecological studies use explanatory inference to test theories and hypotheses. Though commonly used statistical models provide predicted fits to data (e.g., regression coefficients), these within‐sample fits primarily serve to explain mechanisms that may have given rise to the observed data. Explanatory inference provides a means for scientific learning, but the accumulation of evidence for hypotheses and theories across stand‐alone studies can be rather slow (Nichols et al., 2019), and the strict focus on within‐sample model fitting is poorly suited for developing anticipatory policies to deal with ongoing changes in climate, land use, and hydrology (i.e., “global change”; Dietze et al., 2018). In contrast, predictive inference seeks to quantitatively assess model predictions against new observations (Schmueli, 2010). These out‐of‐sample validations are the gold standard for inference in disciplines where vitally important decisions are regularly based on forward‐looking predictions (e.g., meteorology, climatology, epidemiology), but are rarely conducted in ecology (Hooten & Hobbs, 2015).

As global change threatens biodiversity on pandemic levels (Cardinale et al., 2012), some ecologists are striving to become equally as skilled at predicting the future as those in the disciplines listed above (Luo et al., 2011), by the use of either scenario projections or probabilistic forecasts with fully specified uncertainties (Caswell, 2001; Dietze, 2017). The advent of iterative ecological forecasting for guiding near‐term decisions shares many similarities to adaptive resource management (ARM), which has been widely used by applied ecologists (Dietze et al., 2018). For example, temporal applications of ARM have long accounted for multiple sources of uncertainty when evaluating future predictions against newly collected monitoring data as part of a “learn‐by‐doing” process (Holling, 1978; Walters, 1986), with the goal of helping decision‐makers choose the best available action given a specified objective (Williams & Hooten, 2016).

In Section 2 herein, we provide a brief overview of how scientific learning can occur through ARM, and argue that benchmarks (i.e., points of reference) are sometimes needed to assess whether or not the process of ARM is resulting in sufficient learning. Our focus is strictly on model‐based learning about ecological processes through ARM, not on the connection to structured decision making, which has been covered in depth elsewhere (e.g., Runge et al., 2020). More intricate than statistical null hypotheses, we describe in Section 3 that ecological null models are designed to produce an expected pattern in the absence of a hypothesized mechanism, thereby serving as a benchmark for evaluating whether or not the focal mechanism plays an important role in generating empirical data (Gotelli & Ullrich, 2012). They are commonly used by ecologists and evolutionary biologists to learn about the importance of modeled mechanisms in nature (e.g., interspecific competition shaping natural selection and biodiversity; Gotelli, 2001; Harvey et al., 1983). We contend that there is a niche for using ecological null models as benchmarks for learning in ARM, and in Section 4, we demonstrate this utility using a classic case study, harvest management for mallards (*Anas platyrhynchos*) in the North American midcontinent.

## 2 LEARNING THROUGH ARM

A fundamental principle of ARM involves learning about the responses of ecological systems to management actions, and then using that knowledge to inform future decisions. As a consequence, the practice of ARM should reduce uncertainty over time, often in the form of iterated, structured decision making that is connected to model‐based representations of ecological processes (Groot & Rossing, 2011; Williams, 2011). Management actions therefore resemble a form of pseudo‐experiment that should provide more power to isolate key mechanisms compared to pure observation without any intervention, and these insights can be achieved at scales that are not amenable to controlled experiments or interventions (Underwood, 1994; Walters & Holling, 1990). “Active” ARM can result in dual‐control decision policies (Walters & Hilborn, 1978) that attempt to balance the rate of learning with attaining resource management objectives, and thus, managers might temporarily push a system away from optimal resource objectives to elicit stronger mechanistic responses (e.g., alter abundance to a level where density‐dependent feedbacks can be elicited). Such actions help improve understanding of how an ecological system operates, reducing key uncertainties that should increase management performance in the future. In contrast, learning through “passive” ARM may be slower because actions are driven by resource management objectives and not the reduction of uncertainty, which can result in confounding between management actions and ecological processes (Sedinger & Herzog, 2012). Nevertheless, learning might still occur as a by‐product of iterated applications of passive ARM (Schreiber et al., 2004; Williams, 2011).

As applied ecology moved away from the paradigm of comparing single hypotheses against trivial null hypotheses of “no difference,” it shifted into an arguably more powerful paradigm of multiple hypothesis comparison (Burnham & Anderson, 2002; Hilborn & Mangel, 1997), which is also an important aspect of many ARM programs (Nichols et al., 2019). Other applications of ARM use single models and focus on changes in the strength and precision of estimated coefficients for mechanisms hypothesized to affect a focal system as management actions are implemented (e.g., Eaton et al., 2021). Unfortunately, many ecological studies and applications of ARM have abandoned the use of null hypotheses altogether, wherein lies the red herring of thinking that, on their own, model weights, rankings, or parameter estimates portray strong evidence for hypotheses (Guthery, 2008). Inclusion of an appropriate null hypothesis might reveal that all models or variables under consideration fail to improve out‐of‐sample predictive inference. Thus, a strict focus on model comparison and parameter estimation can, in some situations, result in inference being based on the “best of a bad bunch” of models and errors of commission (Arnold, 2010). Such pitfalls can easily occur whenever sufficient benchmarks for evidence are not considered (Scheiner, 2004), or when the ability of a model to adequately represent the data is not properly evaluated (e.g., goodness of fit; Conn et al., 2018). In passive applications of ARM where the focus is primarily placed on meeting objectives, the consequences of these pitfalls are that practitioners can become overly confident in their understanding of system mechanisms (Conn & Kendall, 2004), and satisfaction with the restricted model framework becomes ill‐equipped to indicate when adjustments are needed to meet objectives once the system undergoes substantial change (e.g., ARM in an era when deluge transitions to drought; Nichols et al., 2011).

## 3 ECOLOGICAL NULL MODELS

Traditional null statistical hypotheses parsimoniously represent the expectation that observed data arise purely from random sampling, whereas ecological null models acknowledge important elements of a system (e.g., equilibria, process variance) and are designed to evaluate alternative mechanisms of interest while controlling for some variables that are known to affect the system (i.e., they are more than just an intercept‐only model; Gotelli & Graves, 1996). They represent an intermediate point between null statistical hypotheses and mechanistic models by imposing pattern‐based constraints to preserve important features of observed systems (e.g., basic aspects of presiding knowledge), but are devoid of the focal mechanisms being tested (Gotelli & Ullrich, 2012). Failure to outperform an ecological null model may indicate that a particular mechanism plays no role in the study system, that it has been poorly parameterized, that samples size is insufficient for detecting its effect, or that confounding variables have not been accounted for. Carefully designed ecological null models can therefore provide a guard against errors of commission and omission in observational studies, and are used as benchmarks for evidence in some subdisciplines of ecology (Gotelli & Ullrich, 2012). Most notably, ecological null models are used by community ecologists to expose mechanisms of species interactions involved in species coexistence and patterns of biodiversity (e.g., Adler et al., 2010), and by evolutionary biologists to examine the role of competition in shaping natural selection on trait divergence (e.g., Anderson & Weir, 2021). Though ecological null models are traditionally used in explanatory inference, the same philosophy can be used to increase the rigor of predictive ecological inference (Dietze et al., 2018), and the identification of null models for specific types of data continues to be a topic of inquiry (Molina & Stone, 2020).

Fortunately, applied ecologists, including practitioners of ARM, can look to disciplines such as economics, meteorology, climatology, and epidemiology that have ample experience developing null models to improve predictive inference. In meteorology, for example, intuitive measures of “persistence” (e.g., tomorrow's temperature, wind speed, or precipitation will equal the long‐term average for the day among years, or tomorrow's weather conditions will resemble today's) serve as pattern‐based null models for gauging the improved accuracy of near‐term mechanistic weather forecasts (Silver, 2015). Mechanistic forecasting models must beat models of persistence to be considered as skillful candidates among an ensemble of models for real‐world weather forecasting (Hamill & Juras, 2006). In other words, the null models used in meteorology (and other predictive disciplines) serve as benchmarks for gauging evidence and learning about system mechanisms, and can even include sophisticated patterns similar to the null models used in ecology (e.g., cyclic equilibria, environmental stochasticity; Dietze et al., 2018). The use of null models as benchmarks for evidence and learning can also inspire fields to improve anticipatory predictions, such as iterative forecasting efforts that have resulted in steady improvements in weather and climate forecasting skills over time (e.g., see figure 1 in Luo et al., 2011). Learning is defined as the acquisition of new knowledge or skills (OED Online, 2020), and below we demonstrate why ecological null models should be given greater attention as benchmarks for evaluating learning performance in ARM so that better decisions can be made during an era of rapid global change.

## 4 APPLICATION TO A CLASSIC CASE STUDY OF ARM

### 4.1 Background and methodology

Arising out of decades of contentious stakeholder disagreements about the impact of harvest mortality on waterfowl populations, adaptive harvest management (hereafter AHM) was adopted in 1995 to scientifically guide the process of setting harvest regulations for mallards in the North American midcontinent (*Anas platyrhynchos*; Johnson et al., 1993, 1997; Nichols et al., 1995; Williams et al., 1996). Though just one example, the AHM of midcontinent mallards is widely regarded as an ARM success story in the literature (e.g., Nichols et al., 2019, 2021 and citations therein) because of the annual effort to reduce uncertainty through iterated structured decision making that is connected to competing model‐based hypotheses of how mallard demography operates, which is a process that tends to yield more sustainable management outcomes than reactive decisions (Gerber & Kendall, 2018).

Learning is not an explicit objective in AHM (Johnson et al., 2015), but the passive implementation of AHM is directed at resolving structural uncertainty in the mechanisms that govern mallard population dynamics. It does so by considering four models with contrasting hypotheses about survival and reproduction. Age‐ and sex‐specific annual survival probabilities are modeled as a function of harvest rates according to the additive (*S*_a_) versus compensatory (*S*_c_) mortality hypotheses (Anderson & Burnham, 1976; Burnham & Anderson, 1984). Annual reproductive rate is modeled as a function of annually observed wetland abundance and strong (*R*_s_) versus weak (*R*_w_) density dependence, wherein the functions are based on analyses of historical data collected prior to 1995 (see Table 1; Johnson et al., 1997; U.S. Fish & Wildlife Service, 2019). When combined in a factorial manner, these alternative pairs of hypotheses result in four models meant to encompass structural uncertainty (*S*_a_ *R*_s_, *S*_a_ *R*_w_, *S*_c_ *R*_s_, *S*_c_ *R*_w_). As such, the AHM models should be expected to provide a range of under‐ and overprediction in any given year, with the goal of basing decisions more heavily on those models that consistently predict better than others over time. In 1995, initial weights of 0.25 were assigned to each model (i.e., complete structural uncertainty, conditional on the considered models). As new monitoring data become available, combinations of the survival and reproduction submodels are implemented into a balance equation to forecast population abundance for the following spring (see Table 1). Differences between model forecasts and eventual observations of abundance are implemented in Bayes’ theorem to iteratively update the model weights. Over time, model weights have gravitated toward the *S*_a_ *R*_w_ model that has performed best at 1‐year‐ahead forecasting (72% of weight), *S*_c_ *R*_w_ still receives some support (28% of weight), and both *S*_a_ *R*_s_ and *S*_c_ *R*_s_ received near zero support in 2019 (U.S. Fish & Wildlife Service, 2019).

**TABLE 1:** Balance equation used in AHM of mallards in the North American midcontinent that can incorporate one of two mortality submodels (additive harvest mortality *S*_a_, or compensatory harvest mortality *S*_c_) combined with one of two reproduction submodels (weak density dependence in reproductive rate *R*_w_, or strong density dependence in reproductive rate *R*_s_)

AHM balance equation
$N_{t+1}=\gamma_{S}N_{t}\left(mS_{t,am}+\left(1‐m\right)\left(S_{t,af}+\gamma_{R}R_{t}\left(S_{t,jf}+S_{t,jm}\phi_{f}^{\text{sum}}/\phi_{m}^{\text{sum}}\right)\right)\right)$

AHM submodels	
Model	Features
$S_{t,\text{age},\text{sex}}=S_{0,\text{sex}}^{A}\left(1‐K_{t,\text{age},\text{sex}}\right)$	Deterministic model for age‐ and sex‐specific additive harvest mortality (*S*_a_)
$S_{t,\text{age},\text{sex}}=\left(\begin{array}{cc} S_{0,\text{sex}}^{C} & \text{if}\;K_{t,\text{age},\text{sex}}\leq 1‐S_{0,\text{sex}}^{C}\\ 1‐K_{t,\text{age},\text{sex}} & \text{if}\;K_{t,\text{age},\text{sex}}>1‐S_{0,\text{sex}}^{C} \end{array}\right)$	Deterministic model for age‐ and sex‐specific compensatory harvest mortality (*S*_c_) according to a segmented threshold, whereby harvest mortality becomes additive once it exceeds baseline mortality in the absence of harvest; neither of the AHM survival models account for possible effects of density dependence, habitat conditions, etc.
$R_{t}=0.7166+0.1083W_{t}‐0.0373N_{t}$	Weak density‐dependent model for reproduction (*R*_w_) that also includes an effect of wetland availability
$R_{t}=1.1390+0.1376W_{t}‐0.1131N_{t}$	Strong density‐dependent model for reproduction (*R*_s_); differences between the *R*_w_ and *R*_s_ models were based on the 80% confidence ellipsoid of a best‐fitting linear model to historical reproduction data, and the two points on this ellipsoid with the largest and smallest values for the effect of *N_t_* were used to develop the *R*_w_ and *R*_s_ parameters

Ecological null models	
Model	Features
$N_{t+1}=N_{t}+\varepsilon_{t}$	Null model of population persistence that strips away the vital rate mechanisms in the AHM balance equation, and replaces them with a phenomenological model of temporal process variance around an assumed equilibrium (i.e., $\bar{\lambda }\approx 1$)
$N_{t+1}=\left(e^{r_{\text{eq}}+\beta \cdot W_{t}+\varepsilon_{t}}\right)N_{t}$	Null model with a wetlands predictor that incorporates common knowledge that duck populations ebb and flow with wetland availability, but does so without specifying the vital rate mechanism(s) that may govern such effects, whereas AHM models include such effects for reproduction but not mortality

Parameters	Explanation
$N_{t}$	Currently observed abundance
$N_{t+1}$	One‐step‐ahead forecast of abundance
*m*	The proportion of males in the breeding population treated as a constant (0.52)
$\gamma_{S}$	Constant bias correction factor (0.94) applied to survival to improve forecasts of $N_{t+1}$
$S_{t,\text{am}}$	Adult male survival from year *t* to *t* + 1, modeled as a function of harvest rate according to either the *S*_a_ or *S*_c_ hypothesis, but not estimated directly from contemporary band‐recovery data
$S_{t,\text{af}}$	Adult female survival from year *t* to *t* + 1, modeled as above
$S_{t,\text{jm}}$	Juvenile male survival from year *t* to *t* + 1, modeled as above
$S_{t,\text{jf}}$	Juvenile female survival from year *t* to *t* + 1, modeled as above
$S_{0}$	Baseline survival in the absence of harvest, which is assumed to be different in additive (A) and compensatory (C) models above
$K_{t}$	Harvest rate adjusted for crippling loss (i.e., kill rate)
$\phi_{f}^{\text{sum}}/\phi_{m}^{\text{sum}}$	Ratio of female to male summer survival treated as a constant (0.90)
$\gamma_{R}$	Constant bias correction factor (0.86) applied to reproduction to improve forecasts of $N_{t+1}$
*R_t_*	Predicted reproduction in year *t* (offspring recruitment to fall flight), modeled as a function of *N_t_* according to either the *R*_w_ or *R*_s_ hypothesis and wetland abundance at *t*, *W_t_*, but not estimated directly from contemporary reproduction data
*W_t_*	Wetland abundance at *t*, which is modeled with an autoregressive pattern and temporal process variability (using another submodel not shown here) that facilitates prediction at *t* + 1
$\varepsilon_{t}$	Error term for temporal process variance of population dynamics within one of the two null models
$r_{\text{eq}}$	Average per capita growth rate at equilibrium that equals 0
β	Estimated effect of wetland abundance on population growth rate

Note

See U.S. Fish and Wildlife Service (2019) for details. Briefly, both the survival and reproduction submodels are based primarily on data collected prior to 1995. In 2002, both the survival and reproduction submodels were altered to include bias‐adjustment terms to account for severe discrepancies between model predictions and empirical data, but the basic structure of each submodel remained unchanged. These correction factors were then applied from 1995 onward to improve model predictions, though the correction factors provide ostensible explanations of the data (Runge et al., 2002). For reference, we also provide the two ecological null models presented as benchmarks for learning about the ability of mechanisms contained in the AHM models to provide more accurate predictions of future mallard abundance.

Models can nevertheless make reasonable predictions even when the governing mechanisms are poorly specified (Johnson et al., 2002). For example, because mallard harvest rates are highly correlated with changes in population density, and because density‐dependent natural survival (i.e., survival in the absence of hunting) is not explicitly considered in the four AHM models, support for the additive mortality hypothesis may be an artifact of not including density dependence in survival (i.e., a problem of confounding variables; Conn & Kendall, 2004; Sedinger & Herzog, 2012; Sedinger & Rexstad, 1994; Zhao et al., 2016). This and other factors have spurred the suggestion that the four models currently used in AHM are limiting abilities to learn about the system, which in turn may limit future management performance (Conn & Kendall, 2004; Johnson et al., 2002).

It is always healthy to question whether a model is adequately specified (Box, 1976). A simple ecological null model for gauging the predictive performance (i.e., skill) of mechanistic population models is that of “population persistence,” which is related to the concepts used in meteorology mentioned earlier, and assumes that a population remains at equilibrium where the birth and death mechanisms strike a perfect balance (i.e., $\bar{\lambda }\approx 1$). This would effectively cancel out the vital rate mechanisms in the AHM balance equation (Table 1), leaving a model where the prediction of abundance at *t* + 1 is equal to the observed abundance at time *t* (*N_t_*_+1_ = 1∙*N_t_*, or *N_t_*_+1_ = *N_t_*). Though system persistence is rarely considered as a benchmark for evidence and learning in ARM, an interim strategy for the AHM of mourning doves did consider its utility (*Zenaida macroura*; Sanders & Seamans, 2012). In practice, one might want to consider greater realism by allowing for stochastic variation around an assumed equilibrium with a random walk:

$$N_{t+1}=N_{t}+\varepsilon_{t}$$

where ε*_t_* ~ *Norm*(0, σ^2^). This simple null model of population persistence captures the intuition of a naturalist that waterfowl populations do not radically change from one year to the next (i.e., $\bar{\lambda }\approx 1$). Alternatively, one might consider an ecological null model that adds an effect of wetland abundance at time *t* (*W_t_*) on the population growth rate from *t* to *t* + 1, sans the vital rate mechanisms, since it has long been known that duck populations ebb and flow with wetland availability (Lynch, 1984):

$$N_{t+1}=\lambda_{t}\cdot N_{t}$$

$$\log\left(\lambda_{t}\right)=r_{\text{eq}}+\beta \cdot W_{t}+\varepsilon_{t}$$

where *r*_eq_ is the average per capita growth rate at equilibrium that equals 0, *W_t_* is standardized (mean 0, s.d. 1), ε*_t_* ~ *Norm*(0, σ^2^), and the parameters (*N_t_*_+1_, β, ε*_t_*) are estimated recursively with data up to time *t*.

We compared one‐step‐ahead forecasts at *t* + 1 associated with each of the four AHM models to eventually observed abundances at *t* + 1 for midcontinent mallards between 1996 and 2019. We performed the same out‐of‐sample predictions for a weighted average of the four AHM models, and for our two ecological null models (the population persistence and wetland models; see Supplement for modeling details). We scored each model's forecasting skill across the time series using the root mean square error (RMSE; Dietze et al., 2018). Specifically, we normalized the RMSE by the mean of observed abundances over the time series (NRMSE) to help facilitate comparison of scores among models. The NRMSE balances prediction bias with precision, but it does not measure systematic bias. Systematic bias is a tell‐tale sign of a misspecified model, which we measured using the normalized mean signed difference (NMSD). The NRMSE and NMSD can certainly be measured and evaluated in the absence of an ecological null model, but below we demonstrate how comparisons of these scores with those for a null model can serve as benchmarks for evaluating model‐based evidence and learning about ecological processes in ARM.

### 4.2 AHM forecasting skill

**FIGURE 1:**
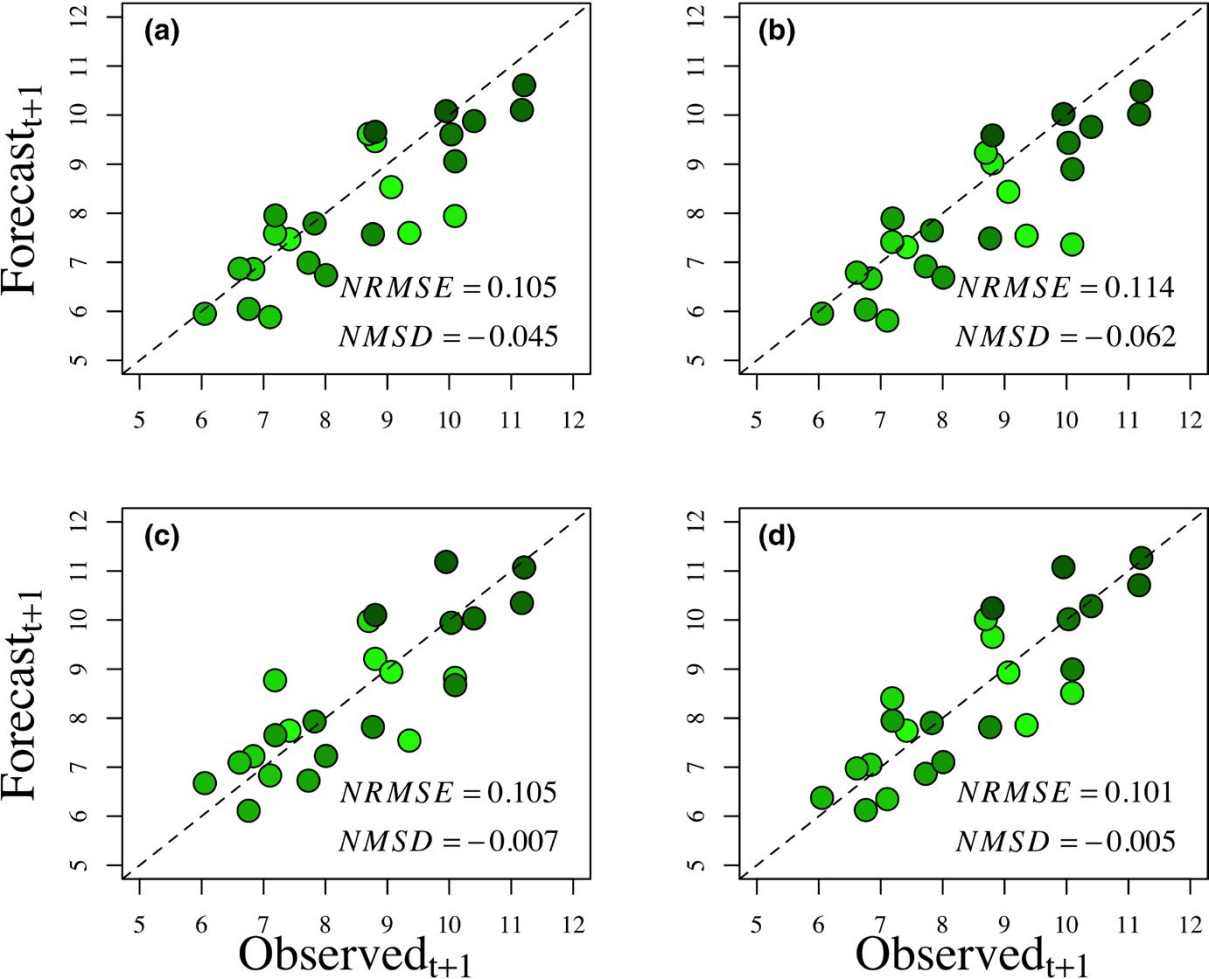
Forecasted mallard abundances (in millions) at time *t* + 1 plotted against the observed abundances at *t* + 1 for the *S*_a_ *R*_w_ (a) and weighted average AHM models (b), compared with the null models of population persistence (c) and that with an additional parameter for an effect of wetlands (d; the other AHM models described in the text are not shown because they currently receive little to no weight, but see Appendix S1 for pertinent results). The expected 1:1 relationships are shown with dashed lines, which are equivalent to the bullseye of a forecasting target. Also provided are the normalized root mean square error (*NRMSE*) and normalized mean signed difference (*NMSD*) for each model. Note that forecasted precisions of the null models are scattered nicely around the targeted relationship (c & d), indicative of unbiased predictions, whereas the tendencies of the AHM models (a & b) are to underpredict observed abundances. Shading of the green circles becomes increasingly darker over time; more recent years have a darker shade

Our ecological null model of population persistence, devoid of any mechanisms, yielded an equivalent NRMSE as that for the *S*_a_ *R*_w_ model, which is currently the top‐weighted model in AHM. This equivalence is not terribly surprising because as complexity is added to models, bias should be reduced while variance increases (Burnham & Anderson, 2002). What is more insightful is that the NMSD of the persistence model was more than six times better than that of the *S*_a_ *R*_w_ model, and eight times better than the AHM model‐averaged predictions. The ecological null model with a wetland predictor had an even better NMSD than the persistence model (Figure 1). Forecasting skills of all other AHM models were notably worse (Appendix S1).

AHM has been a success in many regards because it has gathered disparate stakeholders around a structured decision‐making process, and it has opened people's minds to the concepts of alternative models (e.g., multiple working hypotheses) and learning by doing. Given that the AHM models consistently underpredict observed abundance and cannot surpass the NMSDs of ecological null models, however, there seems to be ample room for improvement as a means to guide science‐based decision making in a changing world. Though systematic biases in the AHM models have been acknowledged (e.g., U.S. Fish & Wildlife Service, 2010), our ecological null models provide the needed benchmark for gauging the severity of systematic bias and inability to surpass null representations of basic knowledge. It therefore seems to be an appropriate time for the AHM community to embrace new model‐based hypotheses and methodologies going forward.

Discrepancies between scientific insight and management practice likely exist in many if not all applications of ARM because the process of structured decision making must also balance trade‐offs with stakeholder desires (Runge et al., 2020; Westgate et al., 2013). Ideas that have been suggested based on scientific studies, but not yet implemented in midcontinent mallard AHM, include the treatment of additive and compensatory mortality as a continuum as opposed to a discrete model choice (e.g., Burnham et al., 1984; Conroy & Krementz, 1990), the incorporation of ecological drivers of survival alongside effects of harvest (Sedinger & Herzog, 2012; Zhao et al., 2018; TVR pers. comm.), flexible parameterizations for the influence of conspecific and wetland densities on fecundity (Specht & Arnold, 2018; Zhao, Arnold, et al., 2019), cross‐seasonal environmental effects on reproduction (e.g., Heitmeyer & Fredrickson, 1981; Osnas et al., 2016, BSS pers. comm.), and individual heterogeneity in demographic performance and vulnerability to harvest (Arnold, 2021; Cooch et al., 2014; Johnson et al., 1984; Lindberg et al., 2013). As in other applications of ARM, stakeholder concerns can impede adaptive considerations of new models in AHM. From a data perspective, however, waterfowl managers could make better use of the monitoring systems already in place and intended to measure changes in reproductive success (age ratios from the Parts Collection Survey; U.S. Fish and Wildlife Service), survival (systematic banding and hunter recoveries; USGS Bird Banding Laboratory), and abundance (Waterfowl Breeding Population and Habitat Survey; U.S. Fish & Wildlife Service, 2021) on an annual or even seasonal basis (Devers et al., 2021). Since 1995, only the latter dataset has been used in AHM (see U.S. Fish & Wildlife Service, 2019), and integrated population models provide one approach to utilizing all of these monitoring data in iterative applications of AHM (Arnold et al., 2018).

Serendipitously, the AHM community has long recognized the limitations of the four models for midcontinent mallards (Johnson et al., 2002; U.S. Fish & Wildlife Service, 2010), and recently highlighted the need to re‐evaluate and update the functional relationships used to predict mallard population dynamics. As part of the double‐loop learning process of ARM (Williams & Brown, 2018), the Mississippi and Central Flyways along with the U.S. Fish and Wildlife Service have been reconsidering all elements of the AHM framework, including deliberations of appropriate harvest management objectives, evaluating alternative regulatory options, and the exploration of Bayesian integrated population models to estimate important population parameters that form the basis for deriving harvest policies. Unfortunately, the COVID‐19 pandemic has impeded the aerial survey of waterfowl abundances that AHM decisions hinge upon (U.S. Fish & Wildlife Service, 2021), and has stalled progress on re‐evaluating components of AHM. Concurrently, a severe drought has recently gripped the Prairie Pothole Region (see referenced CDM & NDMC drought monitors), the core breeding area for midcontinent mallards, further emphasizing the need for AHM to be based on more accurate forecasting models than those used in the past.

## 5 DISCUSSION

The purpose of ARM is not to seek truth with modeling, which is impossible (Box, 1976), but rather to resolve uncertainty about system responses to actions and apply that learning to future decisions in pursuit of management objectives (Nichols et al., 2019). Perhaps because of the need to deal with uncertainty at large scales, and because models are often cast within the context of informing decisions as opposed to that of testing hypotheses, practices of ARM commonly lack the benchmarks used in evidence‐based science (Gillson et al., 2019). Indeed, we searched the literature and found the use of ecological null models or similar benchmarks for evidence and learning in ARM to be rare (see Table 2). Gillson et al. (2019) suggest that concepts used in ARM need to be merged with those used in smaller‐scale practices of evidence‐based science to inform the decision‐making process. The use of ecological null models may very well provide a seamless way to fuse these philosophies for spatial and temporal applications at large scales.

**TABLE 2:** Results from a Web of Science literature search for studies that may have used ecological null models as benchmarks for evidence and learning in ARM (conducted April 28, 2021)

	Hits	Description
*Main keywords*		
Adaptive Management AND (Wildlife OR Fish* OR Marine OR Terrestrial OR Aquatic OR Habitat OR Ecosystem)	6768	An array of studies that formally addressed the ARM process of monitoring, modeling, and decision making (application) to “learn by doing,” many that misapplied the term to “trial and error” management of natural resources (see Westgate et al., 2013), and yet more that did not pertain to ARM at all
*Additional keywords*		
(Null Model OR Null Hypothesis OR Null Expectation)	14	Seven of the 14 studies did not pertain to ARM, three referred to null statistical models (i.e., random outcome), 1 study implemented an ecological null model and referred to ARM in the discussion but was not an explicit study of ARM, 1 used a null expectation within the application step of ARM (i.e., no action) as opposed to a benchmark model for learning about system mechanisms or structure per se (Ketz et al., 2016), one mentioned the need for ecological null models in ARM but did not actually implement them (Linklater, 2000), and 1 fisheries study actually implemented a null model in an ARM context that was emblematic of a persistence model (Staton et al., 2017).
Persistence AND Model AND (Predict* OR Forecast*)	36	35 of 36 studies used the term persistence as a synonym for the viability of an ecosystem, community, population, or species, not as a benchmark model for gauging evidence or learning, one study implemented a persistence forecasting model but did not pertain to ARM (Page et al., 2018)
Benchmark AND Model	24	14 of 24 studies used the term benchmark differently than as a reference model for learning about system mechanisms (e.g., a historical state of a system for gauging change in the state variable), six studies implemented benchmark models for prediction but did not pertain to ARM, one used a benchmark within the application step of ARM (i.e., no action) as opposed to a benchmark model for learning about system mechanisms or structure per se (Hoggart et al., 2014), 1 theoretical study used benchmark models to assess the ability of an agent (manager) to learn via ARM (Lindkvist & Norberg, 2014), and 1 fisheries study actually implemented benchmark models in an ARM context for learning about system mechanisms (Bischi et al., 2013), as well as 1 restoration ecology study (Parasiewicz et al., 2013)

Note

that topical keywords were always included together and that a simple term such as “adaptive management” also hit the more verbose versions such as ARM and adaptive harvest management. Papers that did not address ARM but separately included the terms “adaptive” and “management” were also found by the literature search.

To realize this potential in a rapidly changing world, we suggest practitioners (a) consider agreed‐upon ecological null models as benchmarks for evaluation of learning in their applications of ARM, (b) iteratively track improvements in the predictive skill of ARM models over time (e.g., figure 1 in Luo et al., 2011), and (c) when necessary, use both (a) and (b) to inspire alternative hypotheses and model structures (i.e., a more rapid trigger for double‐loop learning; Johnson et al., 2015). Other fields that are experienced in forecasting and predictive inference have benefitted greatly from each of these practices (e.g., economics, meteorology, climatology, and epidemiology).

Though models of population persistence and simple phenomenological models commonly provide more accurate forecasts of fish and wildlife abundance than more complicated mechanistic models (Adkison, 2009; Ludwig & Walters, 1985; Ward et al., 2014), which is also true in other complex systems such as economics (Hyndman, 2020; Makridakis & Hibon, 2000), a reliance on ecological null models will never result in learning nor can decisions be based on them (because they will typically exclude the parameters informing management decisions). Ecological null models simply provide a benchmark to surpass in the quest to learn through ARM. Fortunately, it should be relatively easy to overcome these hurdles because of the rapid advancement in quantitative methods that can expedite the scientific method. For example, Bayesian hierarchical models readily allow for the decoupling of sampling, process, structural, and driver uncertainties when making model‐based forecasts (Berliner, 1996). Programming tools for quickly assimilating data into model fitting and ecological forecasting are also advancing rapidly (Simonis et al., 2021; Taylor & White, 2020; White et al., 2019), which can expedite learning about the mechanisms that yield sound, scientific forecasts of the future versus those that do not (Luo et al., 2011; Niu et al., 2014). Multiple monitoring datasets can also be leveraged (i.e., fused or reconciled) to improve inference at multiple scales (Arnold et al., 2018; Maunder & Punt, 2013; Pacifici et al., 2017; Zhao et al., 2019; Zipkin & Saunders, 2018), so long as such methods are used carefully (Riecke et al., 2019). Finally, the careful construction of such models can allow for inference regarding the existing types and magnitudes of uncertainty affecting predictions, guiding future research and ARM efforts.

More generally, learning through ARM could be enhanced by encouraging diverse ways of thinking about the modeling and scientific aspects of decision problems. New and creative ideas arise more quickly from a diverse consortium of thinkers contributing to a common topic of inquiry (e.g., Hong & Page, 2004; Woolley et al., 2010). But without inclusion, institutional diversity initiatives may not be sufficient to generate truly diverse contributions to common topics of inquiry (Puritty et al., 2017). Alongside an array of other strategies, incentive‐based grants or competitions could overcome barriers to the inclusion of diverse groups contributing to ARM, as well as other near‐term forecasting enterprises in ecology (Hyndman, 2020; Petchey et al., 2015).

## CONFLICT OF INTEREST

We declare no conflict of interests.

## AUTHOR CONTRIBUTION

**David Koons:** Conceptualization (lead); Formal analysis (lead); Investigation (equal); Methodology (equal); Validation (equal); Visualization (equal); Writing – original draft (lead); Writing – review & editing (equal). **Thomas Riecke:** Conceptualization (equal); Formal analysis (equal); Investigation (equal); Methodology (equal); Validation (equal); Visualization (equal); Writing – review & editing (equal). **G. Scott Boomer:** Conceptualization (equal); Data curation (lead); Formal analysis (equal); Investigation (equal); Methodology (equal); Validation (equal); Writing – review & editing (equal). **Ben S Sedinger:** Conceptualization (supporting); Investigation (supporting); Writing – review & editing (equal). **James S. Sedinger:** Conceptualization (supporting); Investigation (supporting); Writing – review & editing (equal). **Perry J. Williams:** Conceptualization (supporting); Investigation (supporting); Writing – review & editing (equal). **Todd W Arnold:** Conceptualization (lead); Formal analysis (equal); Investigation (equal); Methodology (equal); Supervision (lead); Validation (equal); Visualization (equal); Writing – original draft (equal).

## Supporting information

Supplementary Material

Appendix S1

## Data Availability

All data are in the public domain and are provided by the USFWS at: https://www.fws.gov/birds/surveys‐and‐data/reports‐and‐publications/population‐status.php; https://www.fws.gov/birds/management/adaptive‐harvest‐management/publications‐and‐reports.php; https://www.fws.gov/birds/surveys‐and‐data/migratory‐bird‐data‐center.php
