# Supplementary material for: A niche for null models in adaptive resource management
Source: Ecol Evol. 2022 Jan 13;12(1):e8541. doi: 10.1002/ece3.8541 (PMC8794763; doi:10.1002/ece3.8541)

**Appendix 1** Forecasted mallard abundances (in millions) at time *t* + 1 plotted against the observed abundances at *t* + 1 for the S_a_R_w_ (A), S_c_R_w_ (B), S_a_R_s_ (C), and S_c_R_s_ (D) AHM models compared to the ecological null models of population persistence (E) and that with an additional parameter for an effect of wetlands (F). The expected 1:1 relationships are shown with dashed lines, which are equivalent to the bullseye of a forecasting target. Also provided are the normalized root-mean-square-error (*NRMSE*) and the normalized mean-signed-difference (*NMSD*) for each model. Shading of the green circles becomes increasingly darker over time.


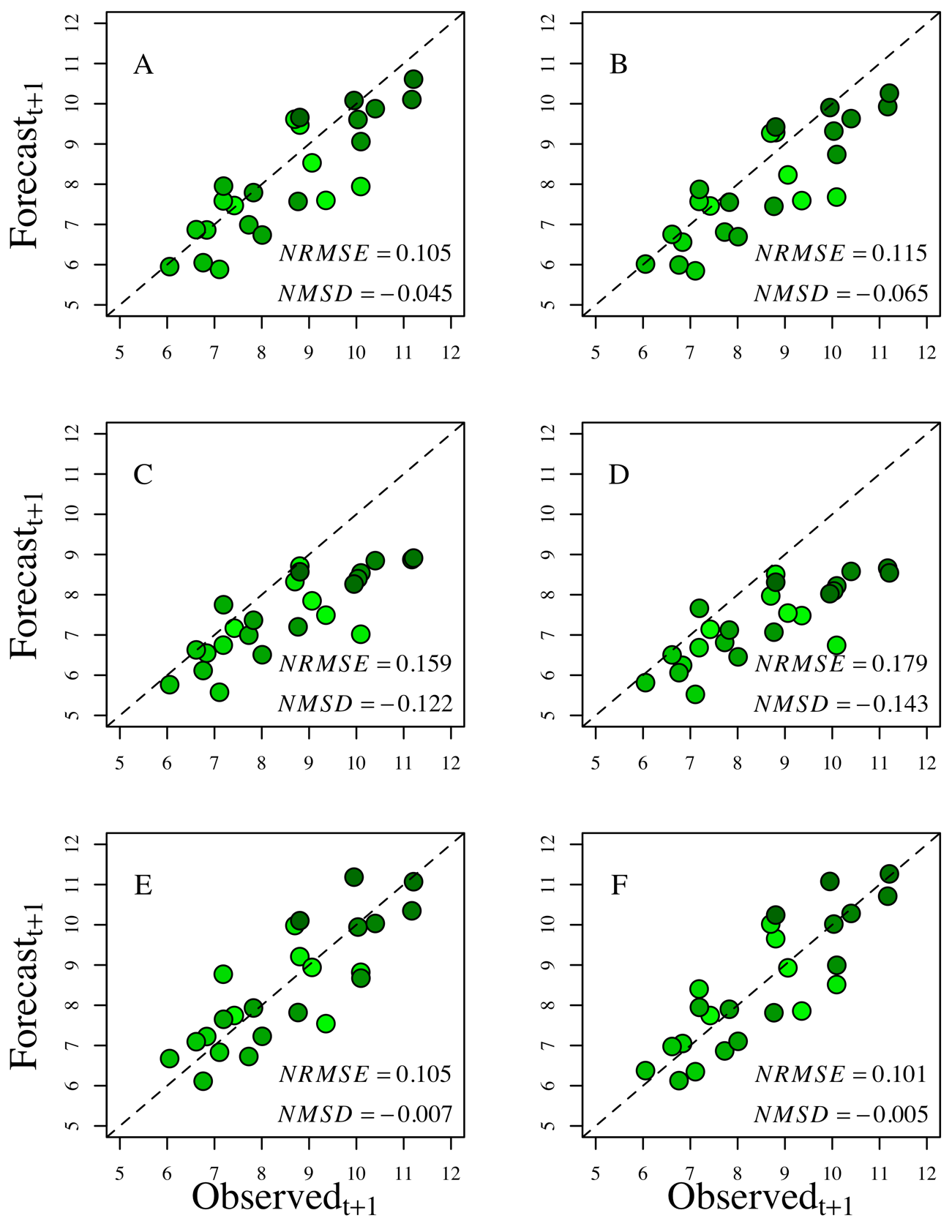

Supplement: Supplementary file 2 — Appendix S1 [file ECE3-12-e8541-s002.docx]
